# Supplementary material for: Real-World Practices of Pentosan Polysulfate Maculopathy Screening in Various Clinical Settings and Practice-Associated Factors
Source: J Clin Med. 2024 Aug 27;13(17):5090. doi: 10.3390/jcm13175090 (PMC11396709; doi:10.3390/jcm13175090)
Supplement: Supplementary file 1 [file jcm-13-05090-s001.zip › jcm-3150601-supplementary.pdf]

Supplementary Table S1. Tests performed for monitoring examinations

Modalities used	Frequency (%)
Funduscopy/fundus photography	6227 (98.0%)
Optical coherence tomography	2891 (45.5%)
Automated visual fields	993 (15.6%)
Fundus autofluorescence	332 (5.2%)
Multifocal electroretinogram	38 (0.6%)
Others	581 (9.1%)
